# A Systematic Review and Qualitative Analysis of Studies Performing Total Knee Arthroplasty (TKA) in Tuberculosis (TB) of the Knee

**DOI:** 10.7759/cureus.57964

**Published:** 2024-04-10

**Authors:** Abin Nizar, Iffath Misbah, Raja Purushothaman, Vasudevan Rajabalaji, Munis Ashraf, Navin Balasubramanian

**Affiliations:** 1 Department of Orthopedic Surgery, Saveetha Medical College and Hospital, Saveetha Institute of Medical and Technical Sciences, Chennai, IND; 2 Department of Radiodiagnosis, Saveetha Medical College and Hospital, Saveetha Institute of Medical and Technical Sciences, Chennai, IND

**Keywords:** recurrence, clinical outcomes, diagnosis, total knee arthroplasty, knee tb, tuberculosis

## Abstract

Tuberculosis (TB) is a widespread global disease that significantly impacts daily life. Skeletal TB comprises about 10-35% of all TB cases. Significant research on the spine and hip exists, but due to the rarity of cases, the management of TB is less explored. Furthermore, exercising the option of total knee arthroplasty (TKA) in TB knees is still in its initial stages. This systematic review aims to identify and comprehend the difficulties associated with diagnosing TB-affected knees, their treatment outcomes, and complications related to TKA. A systematic review of existing English literature retrieved from PubMed, Google Scholar, and Web of Science databases was performed using the PRISMA guidelines. A case series of arthroplasty performed on TB knees included a description of the diagnostic approach, clinical outcome, and complication rates. Moreover, studies involving case series with follow-up functional outcomes were included. The Coleman Methodology was used to assess the quality of the studies. A total of six studies (75 knees) were systematically reviewed in this study. The diagnosis of TB knee is multimodal, with MRI being a reliable tool. Administering anti-TB chemotherapy is essential during the perioperative period. Regarding recurrence, a two-stage TKA has a lower risk of recurrence. It is plausible to state that anti-TB chemotherapy needs to be initiated in the perioperative period to prevent the chances of recurrences. Two-stage TKA is reserved for patients who require soft tissue debridement despite adequate chemotherapy.

## Introduction and background

As of 2022, the incidence of tuberculosis (TB) stands at 127 cases per 100,000 worldwide. The rates are even higher in developing countries (220 in the African region and 211 in South Asian countries) [1]. While pulmonary TB is the most common form of the disease among newly diagnosed cases, a significant number of organs, bones, and joints can also be affected. Undiagnosed TB has a mortality rate of up to 66% [2]. Of all TB cases, osseous TB contributes about 10-35% [2], with the spine being most commonly affected, followed by the hip and knee. The clinical presentation of these infections is quite characteristic, but knee TB has a nonspecific presentation and is often picked up in the late stages of degeneration. The delay in diagnosis would result in a destructed knee, leaving few surgical options [3]. The management of TB knees varies from antitubercular medications to arthrodesis. Over the last few decades, there has been an increasing trend toward total knee arthroplasty (TKA) for TB knee cases [4].

Currently, there is no consensus on the appropriate management of TB knee. Diagnosis of TB knee early is challenging; tubercular tests include biopsy and culture. With the advent of advanced imaging like MRI and CT, these lesions can be detected earlier [5]. Exercising the option of TKA varied among different authors, based on their experience. In addition, the complications following TKA in the TB knee are also unique and different from those of TKA performed in degenerative knees. Furthermore, the timing of surgery, single-stage versus two-stage, type of prosthesis, and cement vs. cementless are some of the major variables that need standardization.

This systematic review aimed to identify and comprehend the difficulties related to the diagnosis of knee TB, the outcomes of its treatment, and the complications associated with TKA. This article also includes a qualitative analysis of the studies that were reviewed.

## Review

Methodology

**Figure 1:**
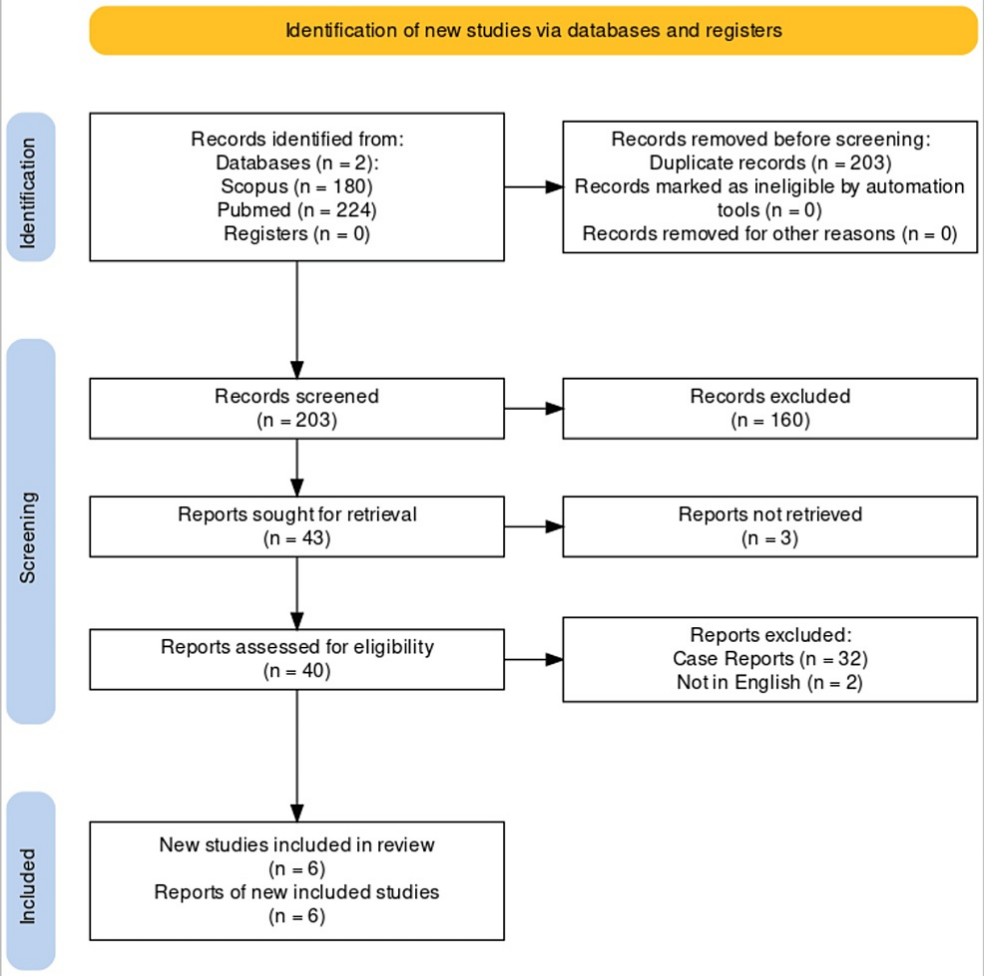
PRISMA flowchart of the selected studies

We have conducted a literature search on PubMed, Scopus, and Web of Science databases using specific keywords (“total knee arthroplasty,” “tuberculosis,” “knee,” “diagnosis,” “outcomes,” and “complications”) to include studies related to TKA in the TB knee (until February 29, 2024). Included were case series of arthroplasty performed on TB knees with the following factors: (a) description of diagnostic approach; (b) clinical outcome; and (c) complication rates. Articles that were isolated case reports (single case study) and not in English literature were excluded. In the initial search, a total of 404 articles were obtained. Out of these, a total of 203 articles were chosen after eliminating any duplicates. Articles that did not meet our inclusion criteria and focused on the TKA in different conditions were excluded. Subsequently, a total of 40 articles written in English and focusing on TKA in TB knees were chosen for abstract screening. After eliminating case studies, articles without follow-up, and duplicates, a total of six articles were included in this systematic review [6-11]. The chosen articles focused on the diagnosis, clinical results, and complications of TKA in the TB knee (Figure 1). Using the Coleman Methodology criteria, the quality of the included analysis was estimated [12].

Results

Among all the studies reviewed, there were a total of 75 knees evaluated. The mean follow-up period among all the studies was 4.4 years (+/- 1.9). The results are presented based on the order of parameters analyzed in this review.

Diagnosis

All the included studies have reported the time at diagnosis (Table 1). For a better understanding of the time at diagnosis, this has been differentiated as a diagnosis made pre-operatively and a diagnosis made post-operatively. In the majority of the studies, almost all the cases have been diagnosed pre-operatively (66/75), whereas 9/75 knees were diagnosed post-operatively. Kim [7], Oztürkmen et al. [9], Habaxi et al. [10], and Zeng et al. [11] have observed that all knees were diagnosed with TB pre-operatively. Su et al. [8] and Eskola et al. [6] have observed few cases of post-operative diagnosis. Apart from clinical evaluation, the diagnostic tests employed were erythrocyte sedimentation rate (ESR), C-reactive period (CRP), acid-fast stain, culture, biopsy, plain radiographs, and MRI of the affected knee joint.

**Table 1:** Study characteristics demonstrating the time of diagnosis of TB and investigations used to aid in diagnosis Pre-op: number of cases diagnosed in the pre-operative period; post-op: number of cases diagnosed in the post-operative period. “+” indicates that the particular investigation was used in the study, while “-” indicates that the particular investigation was not used in the study. CRP, C-reactive period; ESR, erythrocyte sedimentation rate; TB, tuberculosis

Study number	Author	Number of knees	Number of cases diagnosed		Investigations used					
			Pre-op	Post-op	ESR/CRP	Acid-fast stain	Culture	Biopsy	Plain X-ray	MRI
1	Eskola et al. (1988) [6]	6	5	1	-	-	+	-	+	-
2	Kim (1988) [7]	22	22	0	-	-	+	+	+	-
3	Su et al. (1996) [8]	16	8	8	-	+	+	+	+	-
4	Oztürkmen (2014) [9]	12	12	0	+	-	+	+	+	+
5	Habaxi et al. (2014) [10]	10	10	0	+	+	+	+	+	+
6	Zeng et al. (2016) [11]	9	9	0	+	-	-	+	+	+

The laboratory parameters assessed were ESR and CRP. Eskola et al. [6] observed that the ESR was normal in all the cases pre-operatively (6/6). Out of 18 knees, Su et al. [8] had 12 cases with elevated ESR levels. Habaxi et al. [10] noted that all the knees in their study had elevated ESR pre-operatively. Zeng et al. [11] had only four cases (4/9) with elevated ESR and CRP levels. In all the studies, ESR and CRP were performed post-operatively to measure the occurrence of disease reactivation.

The culture of the tissue specimen was performed in all studies except that of Zeng et al. [11]. Here, culture yielded negative results for five out of six knees for Eskola et al. Su et al. reported that none of the 16 knees had yielded a positive culture. The rest of the studies did not mention the results of the culture, which was done.

A biopsy was performed on the tissue specimens only in four of the six studies [7-9,11]. Thirty out of 75 knees were diagnosed pre-operatively [7,8], whereas 29 out of 75 knees were biopsy-proven post-operatively [7,9,11]. Histopathology examination would demonstrate granulomatous lesions, including epithelioid histiocytes, which are surrounded by lymphocytes.

Plain radiographs were taken for all the knees, 75 out of 75, and demonstrated the characteristic signs of bony destruction in TB knees. Studies after 2013 have performed MR imaging pre-operatively and have yielded the diagnoses and extent of soft tissue involvement of TB in all knees.

In summary, among the battery of tests for TB knee, only plain radiographs have been used in all the studies. Regarding the yield of diagnoses, biopsy, plain radiograph, and MRI have yielded the diagnosis of TB in most cases.

Clinical Outcome

The studies assessed patient-reported outcomes and knee range of motion (ROM) after surgery and compared them to the pre-operative state (Table 2).

**Table 2:** Clinical outcomes following TKA HSS, Hospital for Special Surgery Knee Rating Scale; KSS, Knee Society Score; ROM, range of motion; TKA, total knee arthroplasty

Study number	Study	Number of knees	Mean age in years (range)	Mean follow-up in years (range)	Pre-operative ROM	Post-operative ROM	Δ Mean ROM	Outcome score used
1	Eskola et al. (1988) [6]	6	49 (38 to 71)	6.3 (3 to 10)	80° (20° to 100°)	67°	13°	Hungerford and Kenna
2	Kim (1988) [7]	22	53 (17 to 66)	2.7 (2 to 4)	60° (NA)	100° (NA)	44°	HSS
3	Su et al. (1996) [8]	16	58 (27 to 74)	6 (3.4 to 11)	64° (NA)	90° (NA)	26°	KSS
4	Oztürkmen et al. (2014) [9]	12	56 ± 9.8	6.1 ± 1.8	NA	NA	NA	KSS
5	Habaxi et al. (2014) [10]	10	40.6 (22 to 64)	14 ± 0.5 months	NA	95° ± 5°	NA	HSS
6	Zeng et al. (2016) [11]	9	49 (36 to 64)	4.4 (2 to 7)	56° (10° to 90°)	94° (80 to 110)	38°	HSS

In the study by Kim (1988), involving 22 knees with an average follow-up period of 2.7 years, patients had been infection-free for a duration ranging from three months to five years before surgery. In 14 knees out of 22 knees, anti-TB chemotherapy was administered before surgery, and in the rest of the knees (8/22) cases, it was given after surgery. All the TKAs were performed on a single stage. The mean Hospital for Special Surgery Knee Rating Scale (HSS) score rose from 39 points before surgery to 83 points after surgery, indicating improvement. The authors also observed an increase in ROM, from an average of 600 before surgery to 1,000 after surgery. All patients experienced improved ROM, except for one who had repeated infections that required the removal of the prosthesis as well as subsequent arthrodesis, which resulted in low mobility during the final follow-up.

In a study by Su et al. (1996), 16 TKAs were performed on 15 patients, with an average minimum follow-up of 3.4 years. Eight cases received anti-TB chemotherapy before surgery, while the remaining eight were diagnosed with TB post-operatively. All cases underwent a single-stage procedure and received chemotherapy after surgery. The study found that the mean Knee Society Score (KSS) improved from 30.5 to 82.6 points, and the mean ROM increased from 64° to 90° at the final follow-up.

Habaxi et al. (2014) conducted a study involving 10 patients with a mean follow-up of 14 months and found similar results. In all cases, anti-TB chemotherapy was administered before surgery, and a single-stage TKA was performed. The authors observed a significant improvement in the mean HSS score, from 25 to 87 points before and after surgery, respectively, at p < 0.05, and a post-operative ROM of 95 ± 5° at p < 0.05.

Oztürkmen et al. (2014) analyzed 12 patients with knee TB who underwent TKA and were followed up for an average of six years. Four of these patients received pre-operative chemotherapy, while all patients underwent post-operative chemotherapy. The TKA procedures were performed in two stages, with an antibiotic cement spacer used in the first stage followed by implantation in the second stage. The study showed a significant improvement in KSS from an average of 32.4 points before surgery to 83.4 points after TKA (p < 0.05).

Similarly, Zeng et al. (2016) observed improved Harris Hip Scores from 44.8 to 82.7 points at p < 0.05 and knee ROM from 56° to 94° at p < 0.05 in a cohort of nine patients who experienced TKA for knee TB and were followed up for an average of 4.4 years. All patients received post-operative anti-TB chemotherapy for one year. Among the five patients who had not received pre-operative anti-TB chemotherapy, TKA was performed in a single stage.

In contrast, Eskola et al. (1988) conducted a review of six patients with knee TB who received single-stage TKA and were followed up for an average of 6.3 years. Three patients received both pre-operative and post-operative anti-TB chemotherapy. At the final follow-up, using the scale established by Hungerford and Kenna, the authors observed an increase in mean knee scores from 43 to 79.5 points. In contrast to the previous analysis, however, their reported ROM score decreased from a pre-operative score of 80° (evaluated in five of six patients since one knee was ankylosed and minimally mobile) to a post-operative mean of 67°.

To summarize, all the studies with a mean follow-up period of 14 months to 6.3 years showed improvement in clinical outcomes. Improvement in HSS and KSS scores ranged between 36.5 and 62 points from the pre- to post-operative state. Except for one, all studies observed an improvement in the ROM, which ranged between 26° and 44°. Clinical outcomes are illustrated in Table 2, while surgery timing and related factors are discussed in Table 3.

**Table 3:** Timing of surgery, chemotherapy, and staging of surgery A single-stage procedure refers to wound debridement and total knee replacement done in the same setting. A two-stage procedure refers to performing wound debridement initially, followed by total knee replacement after an interval period. ESR, erythrocyte sedimentation rate; TB, tuberculosis; TKA, total knee arthroplasty

Study number	Study	Interval (from TB diagnosis to TKA)	Pre-operative anti-TB chemotherapy	Post-operative anti-TB chemotherapy	Single-stage or two-stage procedures	Cemented or uncemented
1	Eskola et al. (1988) [6]	NA	3 patients received 3 weeks of anti-TB chemotherapy	The same 3 patients received 3 weeks of post-op anti-TB treatment	All single stage	5 cemented and 1 uncemented
2	Kim (1988) [7]	12 months (range: 3 to 12 months)	6 patients for 3 months; 8 patients for 11 to 47 months; and 5 patients received no treatment	All for 18 months	All single stage	4 cemented and 18 uncemented
3	Su et al. (1996) [8]	25.7 months (range: 2 months to 6 years)	8 patients received 2 to 20 months of treatment; 8 were not diagnosed before surgery and received no treatment	1 year	All single stage	13 cemented and 3 uncemented
4	Habaxi et al. (2014) [10]	NA	In all patients for 2 to 4 weeks until the ESR is below 40 mm/H	NA	All single stage	All cemented
5	Oztürkmen et al. (2014) [9]	4 ± 1.5 months	4 patients: 3 presented with septic arthritis, and one had associated pulmonary TB	12 to 18 months in all patients	All two-stage (antibiotic cement spacer followed by implantation)	All cemented
6	Zeng et al. (2016) [11]	3 months to 25 years	4 patients for 3 months; 5 patients received no treatment	All for 1 year	Two-stage in 4 patients who received pre-operative anti-TB treatment; single for remaining 5	All cemented

Complications

The reported complications include recurrence or reactivation of TB, peri-implant loosening, and peri-prosthetic fractures. A recurrence of TB was observed in 10 out of 75 knees, all of which had not received pre-operative anti-TB chemotherapy. Loosening was observed in two cases, and one case of periprosthetic fracture was reported by Su et al. (Table 4).

**Table 4:** Recurrence rate of TB TB, tuberculosis

Study number	Study	Recurrence	Loosening implant-related issue	Periprosthetic fractures
1	Eskola et al. (1988) [6]	1 recurrence, did not receive pre-operative anti-TB treatment	1	Nil
2	Kim (1988) [7]	3 recurrences; those were patients who did not receive anti-TB chemotherapy	Nil	Nil
3	Su et al. (1996) [8]	5 recurrences out of which 4 did not receive pre-operative anti-TB chemotherapy	1	1
4	Oztürkmen et al. (2014) [9]	None	Nil	Nil
5	Habaxi et al. (2014) [10]	1	Nil	Nil
6	Zeng et al. (2016) [11]	None	Nil	Nil

Assessment of Quality

The quality assessment of the analysis was conducted independently by two investigators with the Coleman Methodology criteria, which assigns a score ranging between 0 and 100 points. An outcome of 100 indicates that the analysis has a high level of rigor and minimizes the influence of chance, biases, and confounding factors. The final grade was classified as excellent (85-100 points), good (70-84 points), fair (50-69 points), and poor (<50 points).

The average Coleman Methodology score (CMS) among the reviewed studies was 68.5, indicating that most of the studies had methodological deficiencies. Among the reviewed studies, Su et al. [8], Oztürkmen et al. [9], Zeng et al. [11] and Habaxi et al. [10] had a good score (70-84), while Eskola et al. [6] and Kim [7] had a fair score. However, no studies had a score above 85 (excellent).

The studies reviewed showed major deficiencies in several areas, such as subject selection criteria, study type, rehabilitation protocol description, surgical procedure description, outcome measures, and assessment. The methodological quality of research published in the last decade (average CMS score of 72.33) was slightly better than that of studies published before the year 2000 (average CMS score of 64.2). The list of clinical parameters of all six studies compared is presented in Table 5.

**Table 5:** Quality assessment scores of the studies using the mCMS The mCMS is categorized as excellent (85-100 points), good (70-84 points), fair (50-69 points), and poor (<50 points). mCMS, modified Coleman Methodology score

Study number	Study	mCMS
1	Eskola et al. (1988) [6]	59
2	Kim (1988) [7]	64
3	Su et al. (1996) [8]	70
4	Oztürkmen et al. (2014) [9]	73
5	Habaxi et al. (2014) [10]	70
6	Zeng et al. (2016) [11]	75

Discussion

As of 2020, 10 million people were affected by TB worldwide. This included 1.1 million children. Skeletal TB represents 10-15% of all extrapulmonary TB and 1-2% of all TB cases. Among skeletal TB, the spine constitutes about 45%, the hip 25%, and the knee 8% [13].

In the selected studies, nine knees out of 75 were diagnosed after performing TKA. A definitive pre-operative diagnosis helps in initiating anti-TB chemotherapy early and thereby prevents the chance of recurrence. The clinical features of a TB knee include the insidious onset of swelling, pain, and restricted movements. Laboratory investigations included ESR and CRP, while ESR was used for pre-operative disease activity and CRP was used to assess the response to anti-TB chemotherapy post-operatively. A biopsy of synovial tissue is essential in confirming the histopathological activity of mycobacteria. Before the advent of MRI, surgeons were more reliant on pre-operative biopsies for confirmation of the diagnosis, as evidenced in this review [7,8].

Radiological features are most characteristic, and plain radiographs demonstrate the Phemister triad (juxta-articular osteopenia, peripheral osseous lesions, and joint space narrowing). In all of the studies, plain radiographs were taken, which helped in the diagnosis. However, the accuracy of diagnosing TB knee increased with advanced imaging modalities like the MRI. This was done in studies in the last few decades, and it is to be noted that there were no missed cases of TB knee pre-operatively after the cases were subjected to MRI. The obvious MRI findings of TB inflammation included synovial proliferation, which was hypointense on the T2 sequence, along with periarticular changes. Contrast sequences help in differentiating it from osteomyelitis. The imaging protocol should comprise early post-contrast fat-suppressed T1-weighted sequences in addition to axial fast spin echo T2-weighted, coronal short tau inversion recovery, coronal spin echo T1-weighted, sagittal proton density with fat saturation, and axial DESS sequences [14]. Hence, clinical suspicion with curated imaging is imperative to clinch a diagnosis of TB knee pre-operatively.

Anti-TB chemotherapy was initiated pre-operatively in all the studies. However, the duration of treatment was diverse. Post-operatively, anti-TB chemotherapy was given for at least a year in all the studies. Therefore, it highlights the need to initiate chemotherapy for disease eradication.

Previous studies have shown that TKA may be done in patients with advanced tuberculous arthritis, but there are varying opinions regarding the timing of surgery. The interval between the active infection and operation in our case series ranged from three months to 25 years. Kim [6] proposed a quiescent period of one year to avoid recurrence, noting that cases with a quiescent stage of only three to four months had a higher risk of reactivation. In contrast, Oztürkmen et al. believed that TKA can be performed previously in cases of recent TB onset, and many investigators even argued that active infection must not be incompatible with arthroplasty. Periprosthetic joint infection can be treated successfully with antitubercular therapy alone, a combination of medicine and revision, surgery, or even arthrodesis, despite the risk of reactivation [15-18].

The clinical outcome in the studies was measured using functional outcome scores such as the HSS, KSS, and Hungerford and Kenna scoring systems. The mean follow-up duration was from two years to 11 years. All the authors except Eskola et al. [6] noted an improvement in post-operative ROM and outcome scores. The case series from Eskola et al. [6] demonstrated a decrease in ROM and reduced functional outcome. Some of the factors that caused this result could be the reactivation of TB as chemotherapy was initiated in some cases and the type of prosthesis used (first-generation TKA systems, which were available at the time).

The studies analyzed whether a single-stage or two-stage TKA should be performed. In the study by Oztürkmen et al., all 12 patients underwent a two-stage TKA, while Kim [7] found that only one of the 22 knees underwent a two-stage TKA. Zeng et al. [11] performed nine TKAs, and four patients with high inflammatory biomarkers underwent TKA in two stages, while the remaining five patients with normal inflammatory biomarkers received a one-stage TKA. Su et al. [8], Eskola et al. [6], and Habaxi et al. [10] carried out all events in a one-stage manner. The studies that recommended two-stage surgery had a shorter interval between diagnosis and the first surgery, while studies advocating for single-stage surgery had a one-year or more interval between diagnosis and surgery. During a two-stage operation, thorough debridement is performed in addition to a tissue biopsy to confirm the presence of a current infection, and implantation of an antibiotic-cement spacer can be done as established by Oztürkmen et al. The authors also recommend a six-month gap between the two phases to reduce knee stiffness after the definitive implantation.

The complications included reactivation of TB, periprosthetic loosening, and periprosthetic fracture. Of note, which is beyond the scope of this review, was the incidence of TB knee post-TKA. There are numerous case reports and case series highlighting the development of TB following TKA (prosthetic joint infection). Also, there have been cases of TB joint infection with an incidence of mixed infection with Brucella [19]. The limitations of this study were heterogeneity among the data and a small sample size.

## Conclusions

Given the global impact of TB, evidence is still scanty in TKA for TB knees. With the current review, it is plausible to state that anti-TB chemotherapy needs to be initiated in the perioperative period to prevent the chances of recurrences. Two-stage TKA is reserved for patients who require soft tissue debridement despite adequate chemotherapy. The outcomes following TKA in a TB-affected knee seem to be promising with careful patient selection. Despite the existing evidence, further multicenter, long-term studies are required to frame consensus and treatment protocols.
